# The Objections to the Metric System

**Published:** 1880-06

**Authors:** C. W. Erwin

**Affiliations:** Allendale, S. C.


### THE OBJECTIONS TO THE METRIC SYSTEM.

By C. W. ERWIN, Allendale, S. C.

*Editor Atlanta Medical and Surgical Journal:*—As you have always been on the alert for the advancement of medical science, and not susceptible of being carried away by mere novelty, I beg space for a word of protest against what seems to be a useless innovation; a thing not absolutely new, but copied from the French and accommodated to our acceptance. Indeed, it would appear that some of our pharmaceutic associations and a few of the best authors have already adopted it—I allude to the metrical system. This I am satisfied is not only more complicated and more difficult to understand than the common system, but is decidedly less accurate, and at the same time subjects the practitioner and druggist to a greater liability to mistake. To say nothing of the useless employment of Greek and Latin terms of quantity and measure, such as Decimeter, Hectometer and Kilogramm, etc., adopted from language with which but a comparatively small number of American physicians are familiar, there are many objectionable features.

We know of but one advantage claimed for the new system worthy of notice, viz.: It is said to be easier to calculate. This is a mistake. It is true decimals are substituted for vulgar fractions, but just think for one moment of the immense mental worry and fatigue in reducing fractional cubic feet to their respective places in the table. In the reduction the mind is lost, and has no appreciation of the quantity as it is expressed.

No system should take precedence of the old that is not, 1st, as accurate; 2d, as easily and quickly calculated; 3d, does not express weight, quantity, bulk or measure present to the mind as rapidly and, as nearly as may be, the exact appreciation of the quantity, weight or measure expressed by the numbers.

It is not only necessary to calculate by arithmetic the vulgar or decimal fractional part of any unit of measure, but when the answer is called, the mind should grasp the idea of the actual quantity or bulk expressed by figures.

Now we contend that the lower the numbers, and fewer the figures, the more readily the mind takes in the idea. For this reason experience has taught us when figures expressing quantity, weight or measure became too numerous it is best to resort to lower figures of a higher denomination.

The mind may more easily grasp the actual quantity of ten bushels, one pint and one gill, than the same quantity expressed in gills (2565). Take for example:

| Remedies. | Old System. | | New System. |
|---|---|---|---|
| Gum Opii. | Gr. ½ to ii. | = | 0.03 to 0.12 gms. |
| Aconitia, | Gr. ½ 00 to 1-50 | = | 0.0015 to 0.0010 gms. |
| Ferri Chlor., tinct. | Gtts. x to xxx | = | 0.60 to 1.80 C.C. |

The gramm does not run long enough. To be accurate, figures must be so increased that the mind is lost in the estimate, and to avoid this difficulty, the Decimal expressions, as given in dose book of Met. Club., but rudely approximate, while they differ, as is acknowledged, from other posological tables, making a confusion worse confounded.

In the dose book to which we have refered, the equivalent of one grain is put down as 0.60 gramms, and the equivalent of one drachm as 4 gramms, whereas it should be 3.60 gramms, and the ounce 32 gramms, where it should be 28.80 gramms. Here is not only inacuracy, but a useless tax on the memory, and a waste of calculations.

No doubt four-fifths of the physicians of the United States will concur with us in the statement that the system, so far as it has been adopted, should be speedily abandoned, and we think it is quite time that our Medical Journals should be calling attention to its evident demerits.

